# Effective removal of anionic textile dyes using adsorbent synthesized from coffee waste

**DOI:** 10.1038/s41598-020-60021-6

**Published:** 2020-02-19

**Authors:** Syieluing Wong, Nawal Abd Ghafar, Norzita Ngadi, Fatin Amirah Razmi, Ibrahim Mohammed Inuwa, Ramli Mat, Nor Aishah Saidina Amin

**Affiliations:** 1 School of Chemical and Energy Engineering, Faculty of Engineering, Universiti Teknologi Malaysia, 81310 Skudai, Johor Malaysia; 2 Department of Industrial Chemistry, Kaduna State University, Kaduna, Nigeria

**Keywords:** Pollution remediation, Chemical engineering

## Abstract

Adsorption of Reactive Black 5 and Congo Red from aqueous solution by coffee waste modified with polyethylenimine was investigated. The removal percentages of both dyes increased with amount of polyethyleneimine in the modified adsorbent. Characterization revealed that polyethyleneimine modification improved the adsorbent surface chemistry, while slight improvement of adsorbent textural properties was also observed. The adsorbent’s excellent performance was demonstrated by high removal percentages towards the anionic dyes in most experimental runs. The modelling result showed that anionic dyes adsorption occurred via monolayer adsorption, and chemisorption was the rate-controlling step. The adsorbent possesses higher maximum adsorption capacity towards Reactive Black 5 (77.52 mg/g) than Congo Red (34.36 mg/g), due to the higher number of functional groups in Reactive Black 5 that interact with the adsorbent. This study reveals the potential of adsorbent derived from coffee waste in textile wastewater treatment. Furthermore, surface chemistry modification is proven as an effective strategy to enhance the performance of biowaste-derived adsorbents.

## Introduction

Rivers are one of the most significant features of the earth’s landscape. They carry the surface water, together with nutrients, in the sediment areas to the oceans. Rivers are also the significant sources of drinking water and marine life which is a source of food to human and animals. In addition, rivers are the important resource in modern human society, in view of their roles in tourism, hydroelectricity generation etc. Unfortunately, irresponsible individuals and industries also use rivers as convenient locations for disposal of unwanted materials and chemicals. Direct discharge of pollutants from various sources into the water bodies without adequate treatment is reported as the main threat to water security. Dye industry for instance is identified as the tenth most polluting industry to the water in rivers, as 17–20% of the industrial water pollution is contributed by the textile dyeing and treatment^1^. As a result of their wide usage, approximately 5,000–10,000 tons of dyes are released into the waterways annually^2,3^. Congo red (CR) and Reactive Black 5 (RB5) are among the most commonly used dyes since their inception. The molecules of these dyes carry negative charges when dissociated in water, thus the dyes are termed anionic dyes. When discharged directly into surface waters, these dyes prevent the penetration of sunlight into water (which is essential for photosynthesis of aquatic plants), and pose health threats to the aquatic ecosystem and the population surrounding the river. Therefore, proper treatment of textile wastewater is essential to protect the environment and ecosystem.

Adsorption is a well-developed method for dyes removal from the wastewater, due to the process simplicity and lower cost involved compared to other processes^4^. Development of adsorbents from various biomass wastes as replacement of commercial activated carbons further adds to the cost effectiveness of the process^5,6^. Recent studies also revealed the potential of surface chemistry modification in enhancing the adsorbents’ performance^7^. Such modification can be achieved via treatment with chemicals (especially acids and bases) as well as introduction of ionic polymer groups onto the adsorbent precursors. The adsorbents developed solely via surface chemistry modification are reported to possess satisfactory performance in removal of dyes^8^ and heavy metals^9^ from the wastewater. These findings make modified biomass an attractive option of adsorbent, considering their economic advantage due to the less energy input during synthesis procedure when compared to the activated carbon preparations, where carbonization and activation steps are necessary.

Coffee waste (CW) is one of the abundant biowastes that could serve as precursors for adsorbent synthesis, due to the heavy coffee consumption by the consumers (estimated to be 8 million metric tons per year^10^). The high performance of several CW-derived adsorbents in removal of pharmaceutical compounds^11^, dyes^12^ and heavy metals^13^ have been showcased recently by several research teams. However, the potential of CW-derived materials in adsorption of anionic dyes are yet to be reported. Meanwhile, the role of polyethylenimine (PEI) in improvement of anionic dyes adsorption have been demonstrated in a great number of studies^14^, due to the significant amount of positively charged amine groups in PEI which can readily bind to the anionic adsorbates. Curiously, the potential of CW modified with PEI as adsorbent of anionic dye has not been reported in literature.

This work reports the adsorption performance of PEI-CW towards CR and RB5. The adsorption performances of adsorbents produced using different PEI:CW ratios towards the anionic dyes were tested, followed by detailed characterization of the selected adsorbent. The effects of contact time, initial dye concentration, temperature, solution pH, and adsorbent dosage on RB5 and CR dyes adsorption onto PEI-CW were investigated, followed by interpretation of the adsorption data from the modelling result.

## Results and Discussion

### Preliminary study

**Figure 1:**
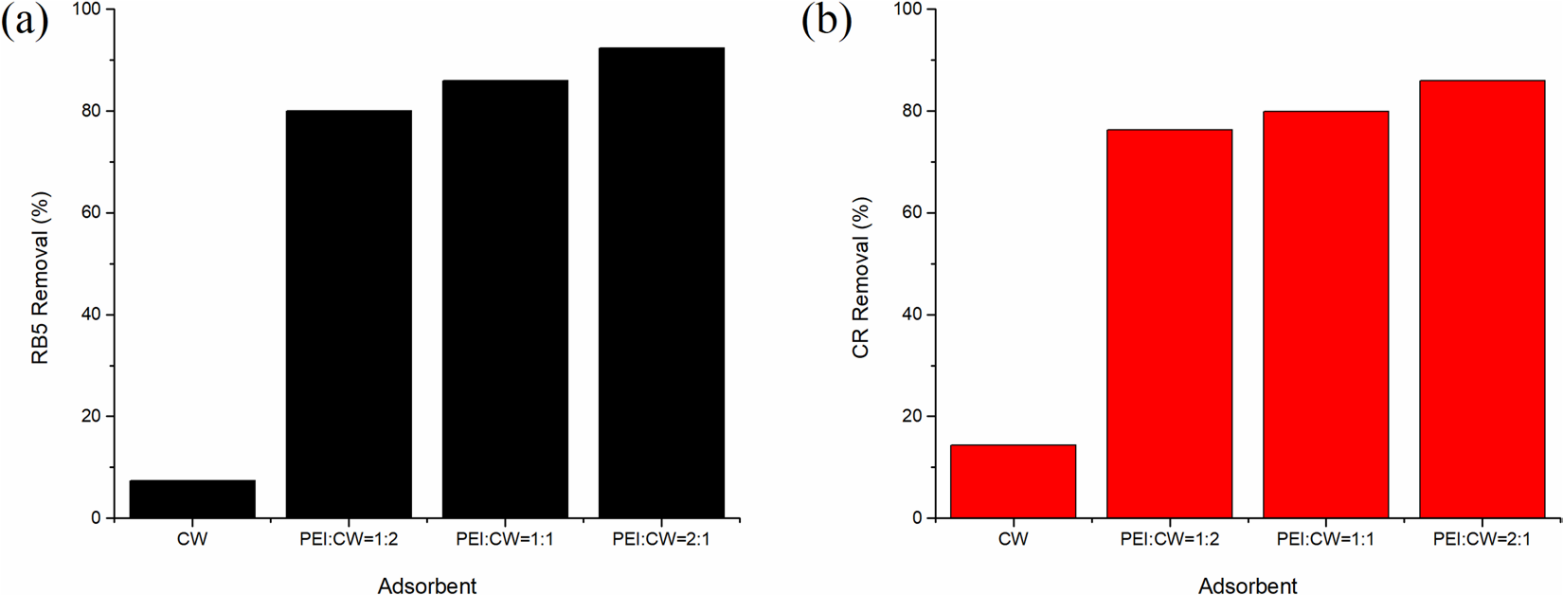
Removal percentages of (**a**) RB5 and (**b**) CR dyes by CW and PEI-CW synthesized using different impregnation ratios.

**Figure 2:**
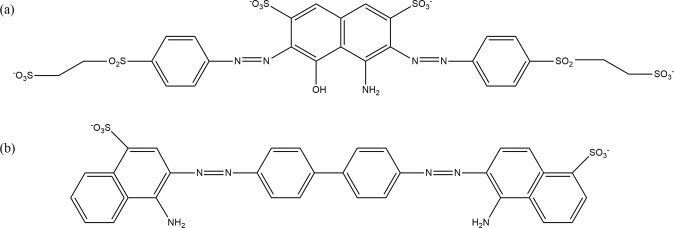
Chemical structures of (**a**) RB5 and (**b**) CR dye molecules.

Numerous research reports indicated that PEI treatment enhances the adsorption capabilities of carbonaceous materials towards ionic dyes despite the lack of pore development^15^. Similar observation was also made in this study, where raw CW showed lower adsorption performance towards RB5 and CR dyes, with removal percentages of 7.42% and 14.35% respectively (Fig. 1), while all PEI-CW adsorbents removed more than 80% RB5 and 76% CR dyes from the solutions under similar conditions. This difference is attributed to the formation of electrostatic attraction between the positively-charged amine groups on PEI-CW surface and the negatively-charged dye molecules^16^. The higher removal of RB5 dye by PEI-CW when compared to CR dye is closely related to the higher charge density of the former adsorbate. A RB5 molecule possesses four sulphonate groups that could interact with the PEI groups via electrostatic attraction and hydrogen bonding, while a CR molecule has only two sulphonate groups (Fig. 2). Therefore, it is more probable for RB5 dye molecules to bind strongly to the adsorbent, leading to a higher removal percentage compared to CR dye. Similarly, the adsorbent with PEI:CW ratio of 2:1 is ranked the best among all the adsorbents tested in this stage, as higher abundance of PEI groups led to higher degree of adsorbent-adsorbate interactions. Therefore, the tested adsorbent was used for the subsequent adsorption study.

### Characterizations of CW and PEI-CW

**Figure 3:**
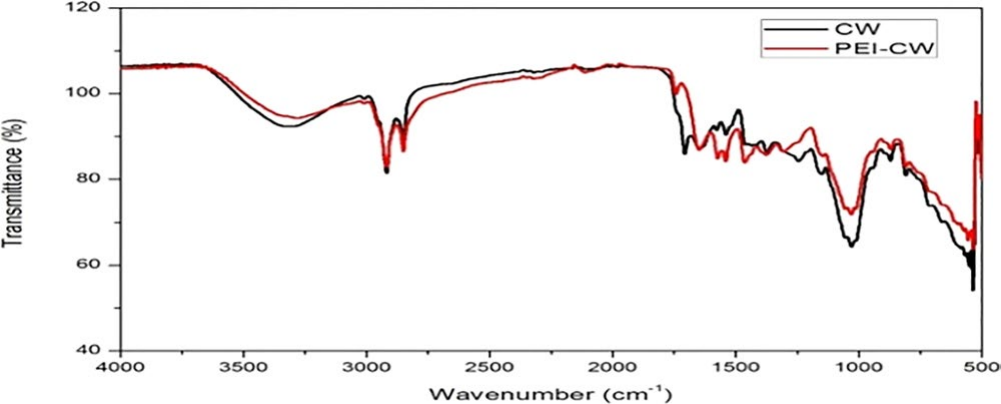
FTIR Spectra of CW and PEI-CW.

The functional groups present on PEI-CW and its precursor are shown in Fig. 3. CW exhibits peaks that correspond to numerous functional groups. The broad band at 3321 cm^−1^ is attributed to the overlapping of O–H stretching and N–H stretching, which signify the presence of hydroxyl and amine groups on the CW surface^5,17^. The peaks at 2918 cm^−1^ and 2851 cm^−1^ are attributed to the aliphatic C–H vibrations of methyl and methylene groups^18^, while another peak at 1707 cm^−1^ is ascribed to the carboxyl C=O stretching vibration^19^. The absorption band at 1645 cm^−1^ denotes the presence of C=N stretching, whereas peaks at 1576 and 1539 cm^−1^ indicate N–H bending on CW^20^. The peak observed at 1460 cm^−1^ indicates the presence of C–H bending on CW^19^. The peaks at 1244 cm^−1^ are related to the presence of C–O group^21^. The broad bands with peaks at 1149 and 1030 cm^−1^ are assigned to C–O stretching from C–O–C bond and C–O–H bond that probably originates from galactomannans polysaccharide’ sugars^21^. The PEI-CW adsorbent produced similar FTIR spectrum with that of CW with few observable changes. The shift in the broad band from 3321 to 3286 cm^−1^ is probably due to the introduction of hydroxyl groups onto CW^16^. Disappearance of the peak at 1707 cm^−1^ was also observed together with the emergence of a new peak at 1744 cm^−1^ which indicates C=O stretching^20^. Three strong peaks are also observed at 1459, 1538 and 1573 cm^−1^ for PEI-CW sample, which correspond to –NH bending^22,23^, C=N bond^23^ and NH_2_ stretching^24,25^ vibrations respectively. Such observation validates the successful crosslinking of PEI onto CW.

**Figure 4:**
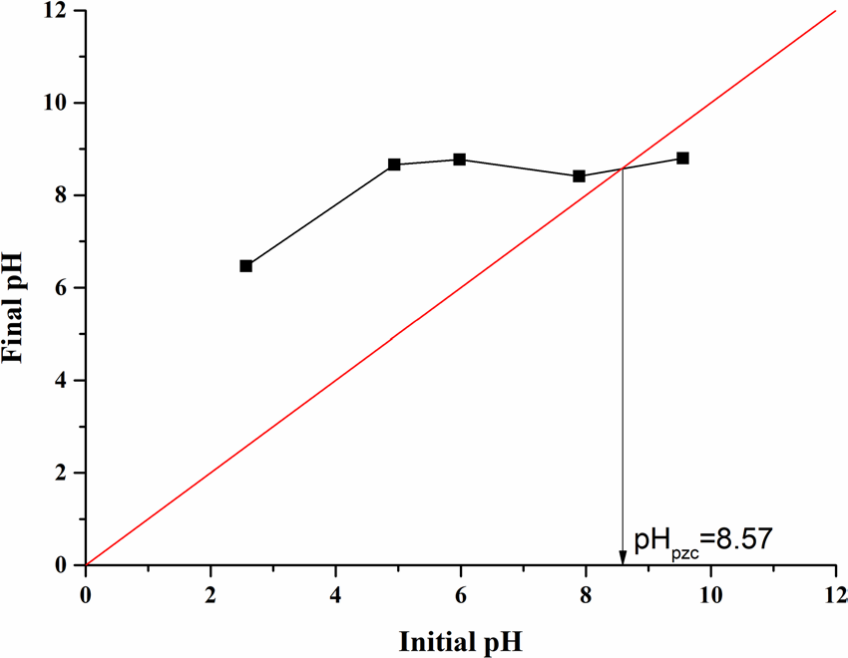
Determination of pH_pzc_ for PEI-CW.

The amounts of acidic and basic oxygenated groups on PEI-CW influenced its interactions with the adsorbates. According to the Boehm titration result (Table 1), PEI-CW contains higher amount of basic functional groups (2.013 mmol/g), while the lactonic acid constitutes 71% of the total acidic functional groups (0.425 mmol/g) on PEI-CW. The presence of lactonic and phenolic groups also explains the peaks observed at 1744 cm^−1^ (corresponding to C=O stretching) and 3286 cm^−1^ (corresponding to the –OH groups) for PEI-CW in Fig. 3. Figure 4 demonstrates a non-linear relationship between the initial pHs and final pHs, which is similar to most absorbents modified with PEI^26^. The high pH_pzc_ (8.57) of PEI-CW indicates the abundant positive charges on the absorbent surface, which can only be completely neutralized under alkaline conditions.

**Table 1:** Boehm titration result and pH_pzc_ for PEI-CW.

Adsorbent	Functional groups (mmol/g)				
	Basic	Lactonic	Phenolic	Carboxylic	Acidic
PEI-CW	2.013	0.300	0.125	0.0	0.425

The textural properties of the adsorbents are shown in Table 2. The BET surface area of CW (1.29 m^2^/g), even after heat pretreatment, is considered low when compared to other coffee waste samples (0.19–11.5 m^2^/g)^13,27^. PEI impregnation slightly increased the adsorbent’s surface area, which is also observed by Saleh, *et al*.^28^ on polyethyleneimine-modified activated carbon. This most probably is related to the repeated washing and drying processes during the modification which removed the impurities on the CW surface and pore system, as illustrated by the increased mesopore and micropore areas of PEI-CW compared to CW. Nevertheless, the textural properties of PEI-CW are far inferior to activated carbons synthesized from biowaste, as illustrated in Table 2. Thus, the role of textural property of the PEI-CW has shown to be negligible during the adsorption process.

**Table 2:** Textural properties of CW and PEI-CW.

Property	CW	PEI-CW	Activated carbon^5^
BET surface area (m^3^/g)	2.115	4.997	1202.8
Mesopore area (cm^2^/g)	1.494	2.056	362.3
Mesopore volume (cm^3^/g)	0.010	0.022	0.37
Micropore area (cm^2^/g)	0.621	2.941	694.1
Micropore volume (cm^3^/g)	0.001	0.001	0.72

**Figure 5:**
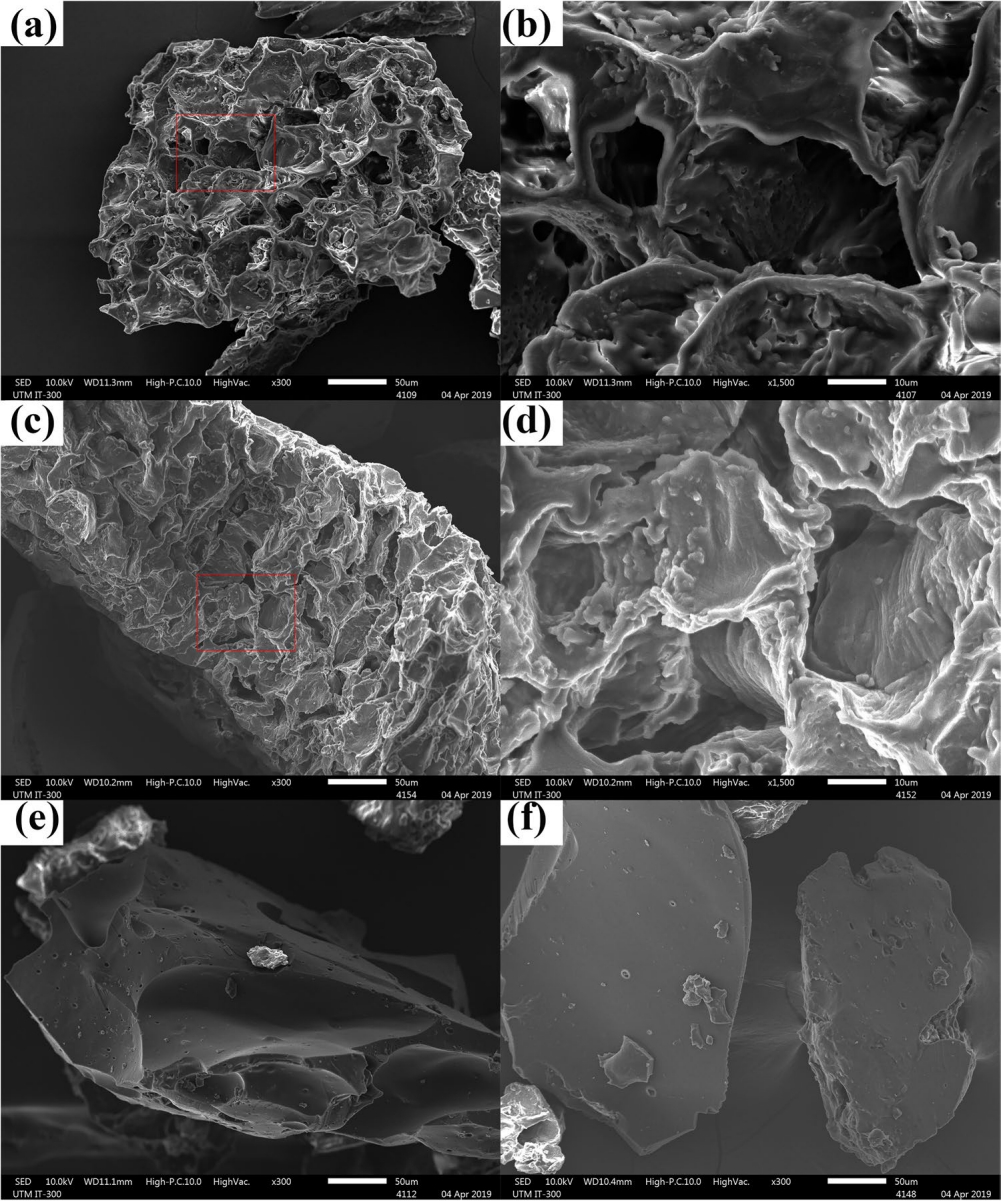
Surface morphologies of CW at (**a**) 300x and (**b**)1500x , PEI-CW at (**c**) 300x and (**d**)1500x, as well foreign particles in (**e**) CW and (**f**) PEI-CW.

The morphologies of CW and PEI-CW are displayed in Fig. 5. The CW exhibits typical three-dimensional carbon structure with rough surface and pore systems (Fig. 5(a),(b)), which is similar to the spent coffee grounds used in other works^29,30^. PEI-CW exhibits denser and smoother structure than CW (Fig. 5(c)) (also observed by Luo, *et al*.^31^), however the pores are still visible (Fig. 5(d)). The presence of foreign particles in CW (Fig. 5(e)) and PEI-CW (Fig. 5(f)) samples was also noticed. These particles possess irregular shapes with sharp edges, thus are attributed to the inorganic particles that are insoluble in water and stable to heat treatment (up to ~100 °C). However, no further investigations on the origins were carried out for these particles.

**Figure 6:**
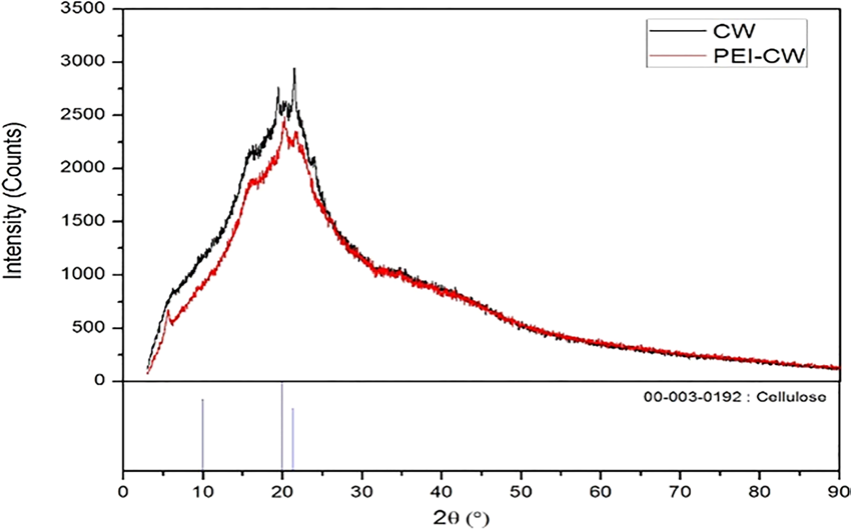
XRD diffractograms of CW and PEI-CW matched with reference peaks of cellulose structure obtained from Internal Chemical Diffraction Data (ICDD).

XRD diffractograms of both CW and PEI-CW (Fig. 6) were matched with the cellulose structure reference acquired from Internal Chemical Diffraction Data (ICDD) with structure code of 00-003-0192. According to Fig. 6, both CW and PEI-CW produced XRD patterns that consisted of amorphous halo with several peaks, thus both structures contain amorphous and crystalline phases, with higher crystallinity being observed in PEI-CW than CW. The crystalline phase is probably attributed to the cellulose in CW and PEI-CW, while hemicellulose and other constituents exhibit amorphous structure due to their susceptibility to chemical attack^32^. The existence of a peak at ~7° is related to increased interplanar distance caused by the larger disorderness produced during the sample modification as observed in methylcellulose samples extracted from the mango seeds fiber^33^ and sugarcane bagasse^34^.

### Batch adsorption study

#### Contact time

**Figure 7:**
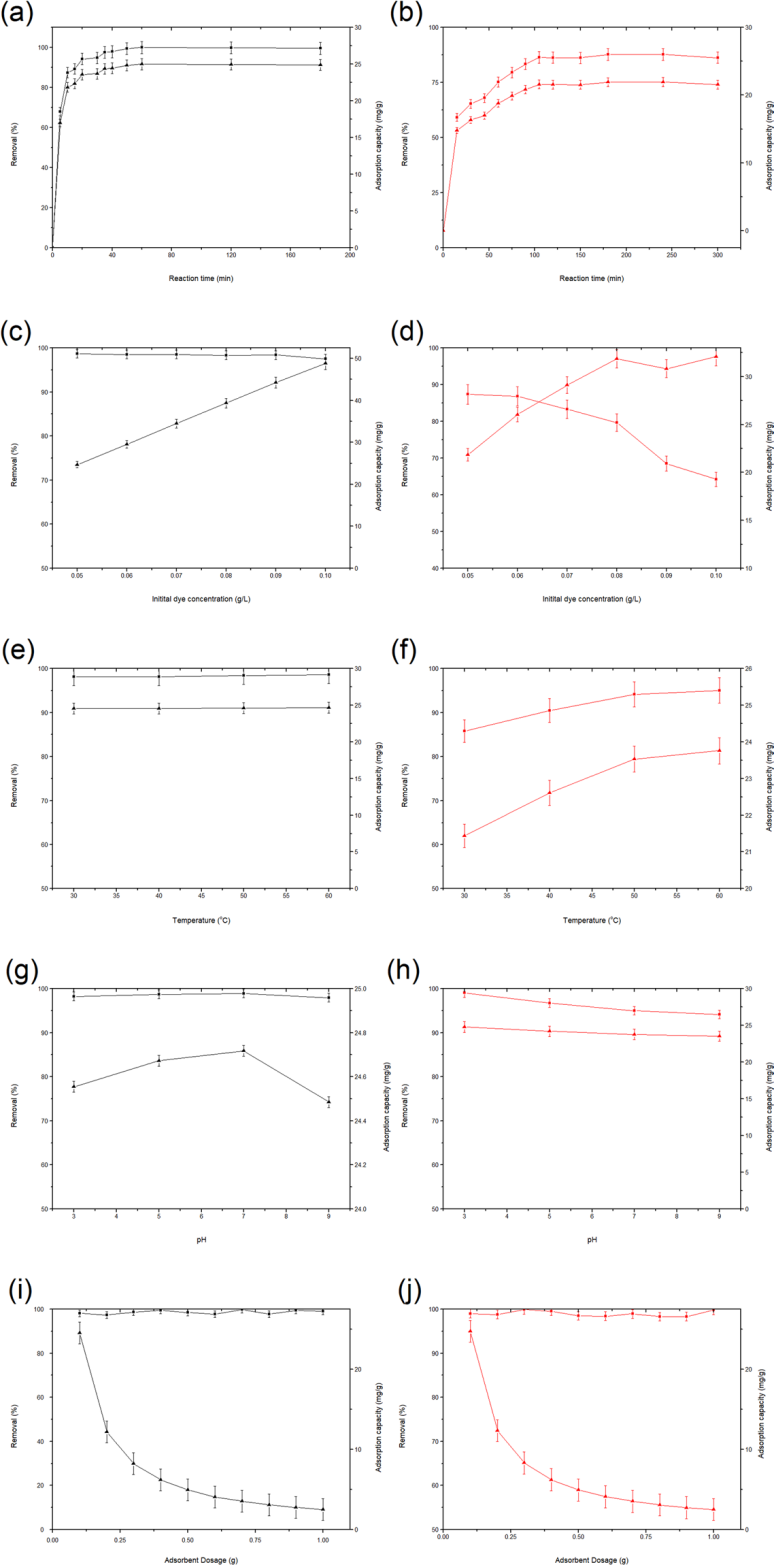
Effects of (**a**,**b**) contact time, (**c**,**d**) initial dye concentration, (**e**,**f**) temperature, (**g**,**h**) solution pH and (**i**,**j**) adsorbent dosage on the RB5 (black) and CR (red) adsorption onto PEI-CW (■ refers to removal percentage; ▲ refers to adsorption capacity).

The effects of contact time on RB5 and CR dyes adsorption onto PEI-CW are shown in Fig. 7(a),(b) respectively. A similar trend is observed for removal percentages of both dyes, where an initial rapid adsorption phase progressed to a slower adsorption phase until a plateau was reached. The maximum dye removal percentages were observed at 50 min (for RB5) and 106 min (for CR) respectively. The changes in adsorption capacities of both dyes by PEI-CW also corresponded to the changes in dye removal percentages. The occurrence of rapid adsorption phase at the initial stage is related to the abundant adsorption sites on PEI-CW that are available for interaction with the dyes molecules in the solution. Following the progression of adsorption process, the number of active sites available for adsorption process decreased, leading to a slower increase in the adsorption rate. The establishment of a plateau indicates the dynamic equilibrium between the adsorption and desorption of the dyes^35^. However, a significant difference could be observed on the removal percentages of RB5 (~99%) and CR (~86%) dyes, corresponding to the adsorption capacities of 24.8 mg/g and 21.0 mg/g, respectively. The higher removal of RB5 compared to CR onto PEI-CW is attributed to the different ionic strengths of the dye molecules as explained in Section 3.1.

#### Initial dye concentration

Fig. 7(c),(d) depict the effects of initial dye concentration on adsorption behavior of RB5 and CR dyes by PEI-CW. High removals of RB5 (98.7%) and CR (87.3%) dyes are observed at low dye concentrations (0.05 g/L). The increase in initial dye concentration equals increase in total amount of dye molecules in the fixed solution volume and adsorbent mass. Thus, more adsorbates could bind to the active sites on the adsorbent at higher dye concentration, resulting in higher adsorption capacities. This explains the increased adsorption capacities of PEI from 24.7 mg/g (at 0.05 g/L) to 48.8 mg/g (at 0.10 g/L) for RB5 dye, and from 21.8 mg/g (at 0.05 g/L) to 32.1 mg/g (at 0.10 g/L) for CR. Meanwhile, competition of more adsorbates for the fixed amount of active sites is also expected at higher initial dye concentration, which results in the saturation of the adsorption sites and dispersion of more dyes molecules in the solution without being absorbed. Subsequently, decreased dye removal percentages to 97.6% (RB5) ad 64.2% (CR) is observed at concentration of 0.10 g/L. A similar trend was also observed in adsorption of CR dye onto *Phoenix dactylifera* seeds^36^.

#### Temperature

Fig. 7(e),(f) depict the effects of temperature on removal percentages of RB5 and CR dyes respectively, with the corresponding adsorption capacities. Significant increase of removal percentage and adsorption capacity of CR dye onto PEI-CW from 85% and 21 mg/g (at 25 °C) to 95% and 23.7 mg/g (at 60 °C) indicates the endothermic nature of the process. The same trend was also observed for CR adsorption onto Chestnut husk-like nickel cobaltite hollow microspheres^37^. Such observation is related to the increased mobility of the dye molecules at higher temperatures, which leads to increased collision and binding of the dye molecules with the adsorption sites on PEI-CW. The endothermic nature is also concluded for RB5 adsorption, as evidenced from the marginal increase in removal percentage (from 98.1% at 25 °C to 98.6% at 60 °C) and adsorption capacity (from 24.5 mg/g at 25 °C to 24.7 mg/g at 60 °C). The reason for the extremely small increase is probably due to the attainment of maximum adsorption of RB5 at 25 °C, in which further increase in the process temperature produced only little effect on the uptake of RB5 dye.

#### Solution pH

The ionization of adsorbates and adsorbent surface charge are influenced by pH of the adsorption system^38^. The effects of solution pH on RB5 and CR adsorption onto PEI-CW are shown in Fig. 7(g),(h) respectively. The removal percentage and adsorption capacity of CR dye on PEI-CW decreased with solution pH from pH 3 (99%, 24.9 mg/g) to pH 9 (94%, 23.5 mg/g). Munagapati, *et al*.^39^ also reported similar observation on CR adsorption onto banana peel powder. As the pH_pzc_ of PEI-CW is 8.57 (as mentioned in Section 3.2), the adsorbent surface is positively charged at pH below 8.57. In acidic medium, the increased H^+^ ion concentration leads to higher protonation degree for the PEI groups on the adsorbent surface, leading to increased electrostatic interaction between the positively-charged adsorbent and the negatively-charged CR molecules, thus the dye uptake^3^. On the other hand, alkaline solution (with high pH) contains more OH^-^ ions, which resulted in increased deprotonation of the amine groups (-NH_2_) on the adsorbent. This change caused decreased electrostatic attraction between the PEI-CW and CR molecules, and hence the adsorption performance^16^. Meanwhile, removal percentage of RB5 dye and adsorption capacity of PEI-CW remains almost constant at around 98–99% and 24.3–24.8 mg/g respectively when the solution pH increased from 3 to 9. This observation shows that electrostatic interaction between adsorbent and RB5 dye plays a more significant role than the solution pH.

#### Adsorbent dosage

The effects of adsorbent dosage on RB5 and CR adsorption are presented in Fig. 7(i),(j) respectively. Almost complete removals of both dyes (98.1% for RB5 and 99.0% for CR) are observed even with 0.1 g of PEI-CW, therefore further increase in adsorbent dosage (to 1.0 g) has little effect on the removal percentages. In contrast, large decrease of adsorption capacity of PEI-CW from 24.5 mg/g (at 0.1 g dosage) to 2.5 mg/g (at 1.0 g dosage) for RB5, and from 24.8 mg/g (at 0.1 g dosage) to 2.5 mg/g (at 1.0 g dosage) for CR was observed. This observation is similar to CR adsorption onto jujube seeds^40^ and RB5 adsorption onto *Macrocystis pyrifera* biomass^41^. Such trend is predictable due to the increased amount of vacant active sites on PEI-CW at higher adsorbent dosage, as the number of adsorbate molecules remains constant. Another possible reason for the observed trend is the inversed relationship between adsorbent dosage and adsorption capacity as shown in Eq. (2)^16^.

#### Adsorption isotherm analysis

**Figure 8:**
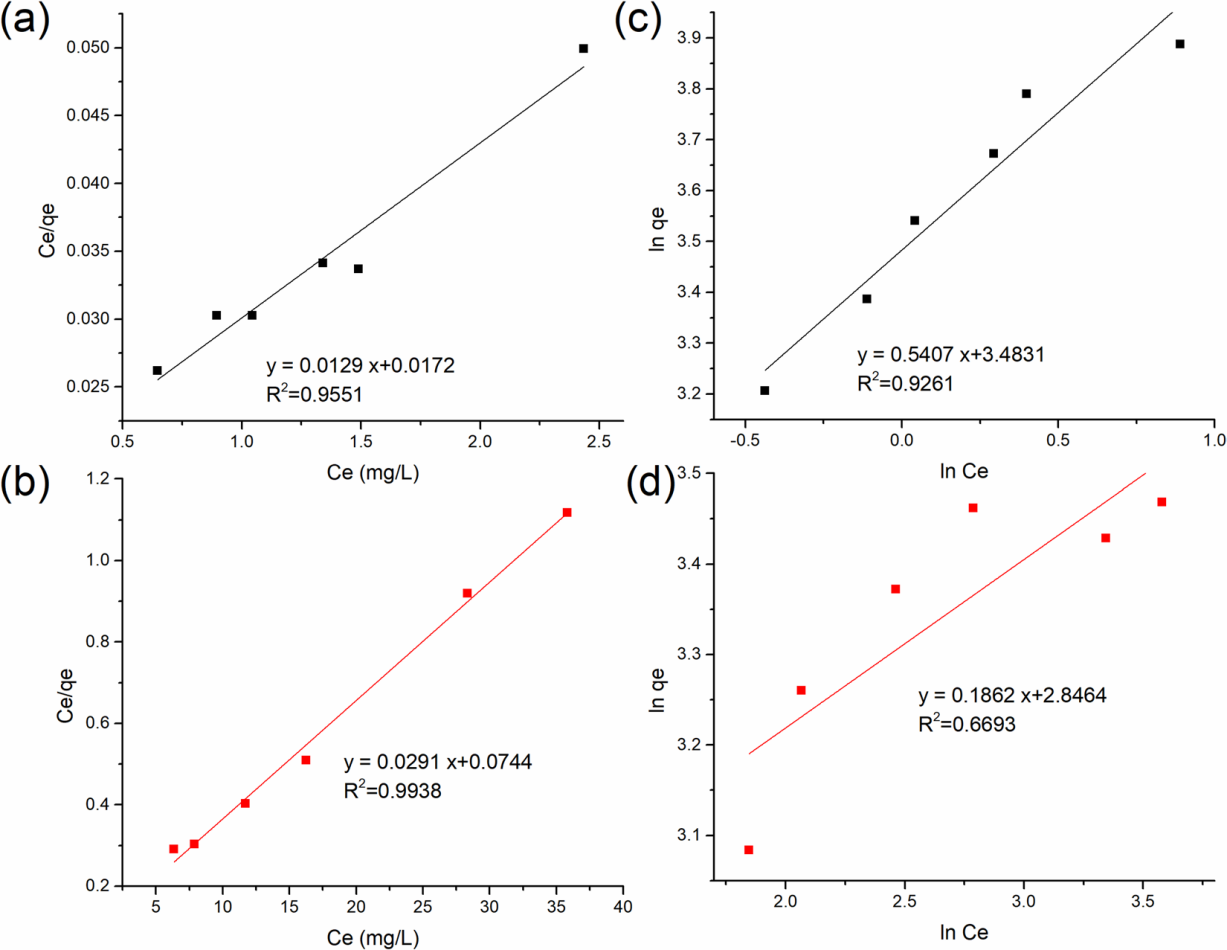
Langmuir isotherm plots for adsorption of (**a**) RB5 and (**b**) CR dyes, as well as Freundlich isotherm plots for adsorption of (**c**) RB5 and (**d**) CR dyes.

The adsorption isotherm analysis was conducted by fitting the adsorption data to Langmuir and Freundlich models, with the details provided in Supplementary Information. The values of linear regression coefficients (R^2^) derived from Fig. 8 along with other adsorption isotherm parameters are expressed in Table 3. Langmuir model is a better fit for the adsorption data of both dyes, as indicated by the higher R^2^ values than those of Freundlich model. Such result indicates the occurrence of monolayer adsorption on the homogeneous surface filled with localized adsorption sites, which was also reported in RB5 adsorption onto canola stalk^42^, as well as CR adsorption onto PEI-wheat straw^8^. The low R^2^ value (0.6693) of the Freundlich linear plot for CR adsorption onto PEI-CW (as shown in Fig. 8(d)) is caused by the non-linearity (1/n < 1) in the adsorption behavior which is typical at high adsorbate concentration, due to the saturation of the active sites on the adsorbent surface. Such a low R^2^ value in linear Freundlich plot is also observed in adsorption of radionuclides^43^ as well as copper (II) ions onto sesame husks, as shown by El-Araby, *et al*.^44^. The maximum adsorption capacity for RB5 adsorption onto PEI-CW is calculated to be 77.52 mg/g, which is higher than that for CR (34.36 mg/g). This is attributed to the higher number of sulfonate groups presented in RB5 molecules than that in CR molecules, which results in stronger electrostatic interaction between the former dye and PEI-CW.

**Table 3:** Adsorption isotherm parameters for CR and RB5 dye adsorption onto PEI-CW.

Dye	Langmuir			Freundlich		
	q_m_ (mg/g)	K_a_ (L/mg)	R^2^	1/n	K_f_ (mg/g)	R^2^
CR	34.36	0.39	0.99	0.19	17.23	0.67
RB5	77.52	0.75	0.96	0.54	32.56	0.93

Recently, a great number of researchers have developed various inorganic adsorbents, including nanoparticles, nanocomposite, graphene-based materials etc, for removal of contaminants from wastewater. A quick comparison (Table 4) shows that these adsorbents possess higher adsorption capacities towards CR when compared to the adsorbents derived from biowaste, including PEI-CW used in this study. Nevertheless, the inorganic adsorbents are inferior to PEI-CW in term of RB5 removal. Such observation is attributed to the ability of PEI-CW in forming the interaction with RB5 molecules with four sulphonate groups.

**Table 4:** Maximum adsorption capacities of various adsorbents towards RB5 and CR dyes.

Adsorbent	Maximum adsorption capacity (mg/g)	
	RB5	CR
**PEI-CW (Present work)**	**77.5**	**34.4**
Dolomite^50^	72.4	229.2
Banana peel powder^39^	49.2	164.6
Hierarchical magnesium oxide (MgO) incorporated fly ash (FA) composite^51^	48.8	—
Zerovalent iron nanoparticles combined with *Macrocystis pyrifera* biomass^41^	39.9	—
Magnetic iron oxide nanoparticle^52^	18.0	—
MoO_2_/CaSO_4_ composites^53^	—	853.5
Hierarchical C/NiO-ZnO nanocomposite^54^	—	613.0
Hydroxyapatite nanoparticles loaded on Zein^55^	—	416.7
Amine-modified *Funalia trogii* biomass^56^	—	193.7
Pineapple plant stem^57^	—	12.0

#### Adsorption kinetics evaluation

**Figure 9:**
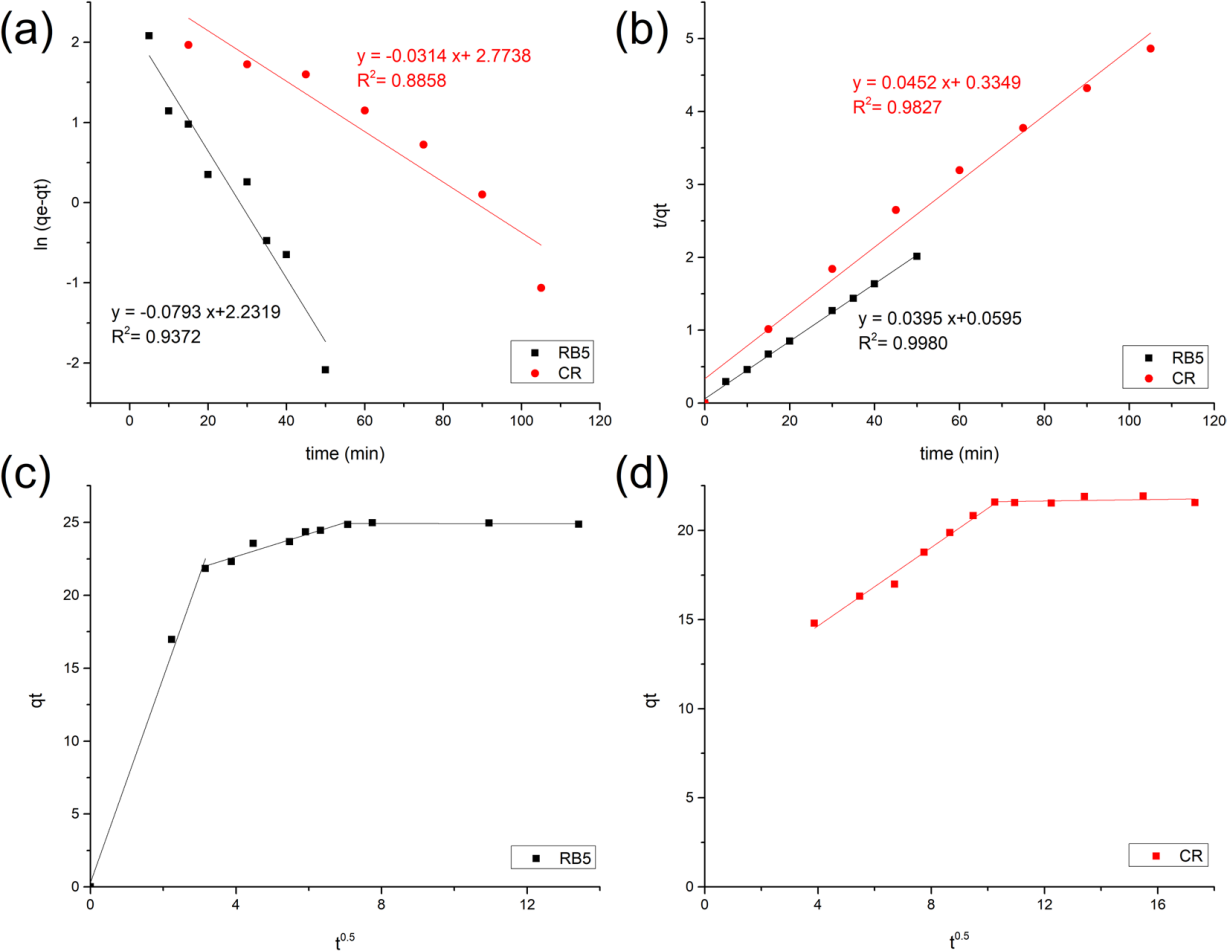
Adsorption kinetic model fitting plots using (**a**) PFO model (for RB5 and CR dyes), (**b**) PSO model (for RB5 and CR dye) and intraparticle diffusion model for (**c**) RB5 dye and (**d**) CR dye.

The kinetic evaluation was carried out by fitting the experimental data to the pseudo-first-order (PFO) and pseudo-second-order (PSO) kinetic models, as described in Supplementary Information. The linear regression coefficient (R^2^), kinetic rate constants (k_1_ and k_2_) and theoretical equilibrium capacity (q_e_) are listed in Table 5. Based on the comparison of the R^2^ values in Fig. 9(a),(b), the adsorption data is fitted better to the PSO than PFO model. This finding indicates that the adsorption of RB5 and CR dyes onto PEI-CW is controlled by chemisorption. Figure 9(c),(d) display the intraparticle diffusion plots for RB5 and CR adsorption respectively. The plot representing CR adsorption consists two straight lines. The first line is related to the external diffusion and significant boundary layer diffusion (as denoted by the large intercept of the straight line). The second line, which represents the occurrence of intraparticle diffusion, is almost horizontal. This observation indicates the low adsorption for such step, due to the lack of pore development in PEI-CW. However, the linear plots representing RB5 adsorption consists three straight lines, indicating the occurrence of three stages. Such observation is probably related to the occurrence of external diffusion, followed by boundary diffusion in two distinctive stages, due to the strong electrostatic interaction between adsorbents and adsorbates.

**Table 5:** Adsorption kinetic parameters for CR and RB5 dye adsorption onto PEI-CW.

Kinetic Model	Parameter	Dye	
		CR	RB5
PFO	k_1_ (min^−1^)	0.03	0.09
	q_e_,_calc_ (mg/g)	18.26	12.98
	R^2^	0.89	0.94
PSO	k_2_ (g mg^−1^ min^−1^)	0.01	0.03
	q_e_,_calc_ (mg/g)	22.13	25.34
	R^2^	0.98	1.00
Experimental q_e_ (mg/g)		21.94	24.98

#### Adsorption thermodynamic analysis

**Figure 10:**
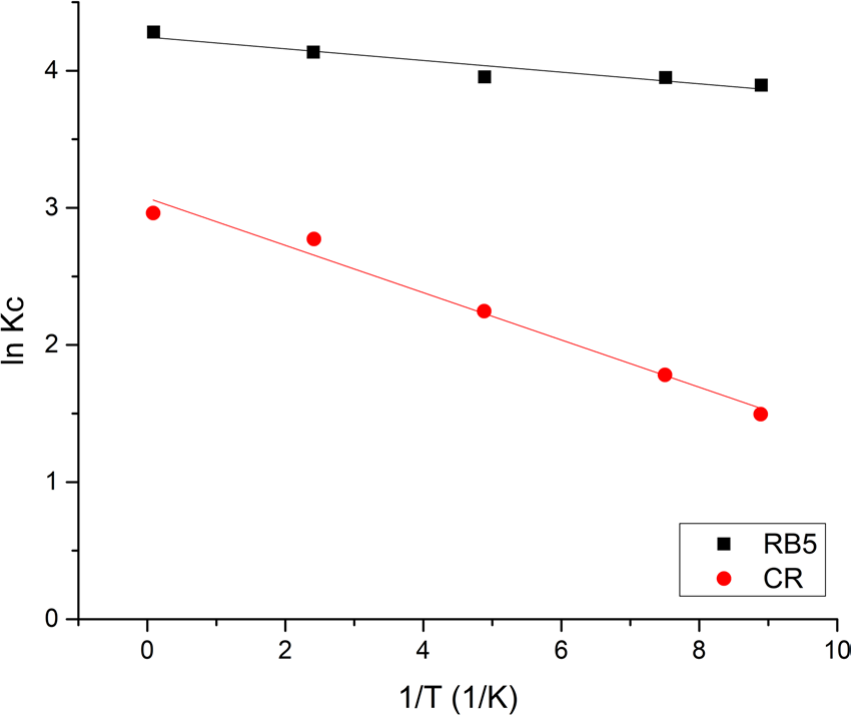
Van’t Hoff plot of CR and RB5 dye adsorption onto PEI-CW adsorbent.

This section reports evaluation of the thermodynamic parameters including changes in Gibbs free energy (ΔG°), entropy (ΔS°) and enthalpy (ΔH°), based on the method described in Supplementary Infomation. Figure 10 demonstrates the Van’t Hoff plots for RB5 and CR adsorption, while the associated thermodynamic parameters are listed in Table 6. The negative ΔG° values which increased with temperature indicate increased spontaneity for adsorption of both dyes. The positive ΔS° values indicate the increased disorder at the interface between the solid adsorbent and the aqueous solution^45^. This further supports the likelihood of occurrence of the process due increased entropy. The positive ΔH° values (35.05 kJ/mol for CR dye, 8.28 kJ/mol for RB5 dye) denote the endothermic nature of the adsorption process for both dyes. Several previous studies on CR dye adsorption onto ground nut shell charcoal^46^ and cationic modified orange peel^47^ as well as RB5 dye adsorption onto banana peel powder^39^ also demonstrated the endothermic nature of the process.

**Table 6:** Thermodynamic parameters for CR and RB5 dye adsorption onto PEI-CW.

Parameter		Dye	
		CR	RB5
ΔG^o^ (kJ/mol)	25 °C	−3.76	−9.66
	30 °C	−4.53	−9.96
	40 °C	−5.86	−10.28
	50 °C	−7.46	−11.08
	60 °C	−8.17	−11.80
ΔH^o^ (kJ/mol)		35.05	8.28
ΔS^o^ (J/mol K)		130.59	59.99

## Conclusion

Following the increasing amount of biomass waste generation, it is necessary to study the conversion of such waste to value-added products, including adsorbents for removal of pollutants from wastewater. In this study, PEI-CW was evaluated as a potential adsorbent in removal of anionic CR and RB5 dyes from aqueous solution. Preliminary study demonstrates that adsorbent prepared using high PEI:CW ratio is more effective in removal of RB5 and CR dyes. Characterization results revealed successful surface chemistry modification for CW, while little change on the textural properties of PEI-CW after modification was also noticed. XRD analysis revealed that both CW and PEI-CW possess crystalline and amorphous phases, due to the presence of cellulose. Batch adsorption study demonstrated that PEI-CW is more effective in removal of RB5 than CR dye, due to the presence of four negatively-charged sulfonate groups in RB5 dye structure which can form stronger electrostatic interaction with the adsorbent. On the other hand, each CR dye molecule only contain two sulphonate groups, thus the adsorptive removal of CR by PEI-CW is less effective. Langmuir isotherm model and pseudo-second order kinetic model fitted well to the data of both CR and RB5 dyes adsorption data. The maximum adsorption capacities of PEI-CW according to the Langmuir isotherm model were found to be 77.52 mg/g (RB5) and 34.36 mg/g (CR) respectively. The thermodynamic analysis suggested that both CR and RB5 dyes adsorption process are spontaneous and endothermic in nature. This study shows that surface modification is a simple yet cost-effective method to enhance the adsorption performance of biowaste-derived adsorbent towards ionic dyes. Potential applications of PEI-CW in removal of other charged/ionic contaminants will be studied in the near future.

## Methods

### Materials

The spent CW used in this study was obtained from a local coffee restaurant in Johor, Malaysia, while the PEI (50 wt% aqueous) and glutaldehyde (GTA, 25 wt% aqueous) were purchased from Acros Organics and Sigma respectively. The used CW was initially washed repeatedly with boiled water to remove soluble compounds. After that, the CW was dried in an oven at 60 °C for 24 hours, followed by sieving to acquire CW particles with size smaller than 500 μm. The PEI impregnation was then performed according to the method described by Jiang, *et al*.^48^ with some modifications. The procedure involved mixing 2.5 g of CW with PEI solution in water bath at 65 °C for 6 hours, followed by addition of glutaraldehyde solution (1% w/v) to the mixture for the crosslinking process. Finally, the adsorbent was filtered, washed with deionized water, and dried at 50 °C for 24 hours. The final adsorbent is termed as PEI-CW. CR and RB5 dyes (Sigma-Aldrich) in powder form were used to produce stock solutions (1,000 mg/L), which were then diluted to produce dyes solutions with desired concentrations. Hydrochloric acid (HCl) and sodium hydroxide (NaOH) solutions, with concentrations of 0.1 M, were utilized to adjust the solution pH.

### Removal of RB5 and CR dyes by CW and PEI-CW

A preliminary study was conducted to compare the adsorption of RB5 and CR dyes onto the PEI-CW prepared using three different PEI/CW (w/w) ratios (1:2, 1:1 and 2:1). The adsorption experiment was conducted under the following conditions: 0.1 g adsorbent dosage, 50 mL dye solution with initial dye concentration of 50 mg/L, pH 7, at room temperature. The adsorption was performed in a rotary shaker with a rotational speed of 200 rounds per minute (rpm) for 120 minutes. The residual dye concentration in the solution after adsorption was determined using UV-Vis Spectrophotometer (Nanocolor^®^ UV/VIS Macherey Nagel) at 497 nm (for CR) and 597 nm (for RB5) respectively. The percentage removals of the dyes in different solutions and the adsorption capacities of PEI-CW were then calculated using Eqs. (1) and (2) respectively. The adsorbent with the highest percentage dye removal was characterized and used in the subsequent adsorption studies.

1 $${\rm{Dye}}\,{\rm{Removal}}( \% )=\frac{{{\rm{C}}}_{{\rm{o}}}-{{\rm{C}}}_{{\rm{e}}}}{{{\rm{C}}}_{{\rm{o}}}}\times 100 \% $$

2 $${\rm{Adsorption}}\,{\rm{capacity}}({{q}}_{{e}})=\frac{({{\rm{C}}}_{{\rm{o}}}-{{\rm{C}}}_{{\rm{e}}})}{{\rm{W}}}\times {\rm{V}}$$

where C_o_ is the initial dye concentration (mg/L), C_e_ is the equilibrium dye concentration (mg/L), V is the volume of the dye solution (L), W is the mass of adsorbent (g) and q_e_ is the adsorption capacity at equilibrium (mg/g).

### Adsorbent characterizations

The changes in surface functional groups on CW after PEI treatment were investigated using Fourier Transform Infrared (FTIR) Spectroscopy (Nicolet IS5, Thermo Fischer Scientific) coupled with Attenuated Total Reflectance (ATR) in the wavelength range of 400–4000 cm^−1^. The instrument was integrated with OMNIC 8.3.103 software for peak analysis. The surface chemistry of PEI-CW was investigated using Boehm titration as demonstrated by Wong, *et al*.^35^. In brief, the amounts of acidic (carboxylic, phenolic and lactonic) functional groups present on the adsorbent surface were calculated based on the amounts of different bases (NaHCO_3_. Na_2_CO_3_ and NaOH) that reacted with the adsorbent, while the amount of basic functional groups on the adsorbent surface was determined via neutralization with HCl. The point of zero charge (pH_pzc_) of PEI-CW was also determined via solid titration method. A fixed amount of adsorbent (0.15 g) was added separately to five Schott bottles containing 50 mL NaCl solution (0.01 mol/L) with pH values ranged from 2 to 12 (by adjustments using 0.1 mol/L HCl and 0.1 mol/L NaOH solutions). The bottles were shaken at 150 rpm to allow equilibrium to establish, then the final pHs of the filtered solutions in the bottles were measured and plotted against their initial pH. The pH_pzc_ value was represented by the intersection point between the straight line y = x and the plot. Boiled distilled water was used for Boehm titration and pH_pzc_ determination to minimize the effect of atmospheric CO_2_ in the solution pH. The effect of PEI modification on the surface morphology of the adsorbent was studied using a variable pressure scanning electron microscope (SEM) (JSM-IT 300LV, Jeol). Nitrogen adsorption/desorption was performed on the samples at 77 K using BET Surface Area and Pore Volume Analyzer (Surfer, Thermo Scientific) to determine the textural properties of CW and PEI-CW. The crystallinities of the adsorbent samples were determined using X-Ray Diffractometer (Smartlab, Rigaku) with Cu tube (λ = 1.54 Å) based on the method described by Wang, *et al*.^49^. The radiation was carried out at 40 mA and 44 kV within the range of 0° < 2θ < 90^o^. The spectra of the adsorbents were compared with the cellulose structure from Internal Chemical Diffraction Data (ICDD).

### Batch adsorption study

The adsorption experiment was conducted to study the effect of contact time (0–180 minutes) on removals of RB5 and CR under the following conditions: 0.1 g PEI-CW, 50 mg/L dye concentration with the solution pH 7 at room temperature in a rotary shaker with a rotational speed of 200 rpm. The residual dye concentrations of the solutions were then analyzed using UV-Vis Spectrophotometer as described in Section 2.2. Similar experimental procedure was also used to investigate the influence of the initial dye concentration (50 mg/L–100 mg/L), temperature (25 °C–60 °C), solution pH (3–9) and adsorbent dosage (0.1 g–1.0 g) on the adsorption process.

## Supplementary information

Supplementary Materials.

## Data Availability

The datasets generated during and/or analyzed during the current study are available from the corresponding author on reasonable request.
