# Supplementary material for: Effective removal of anionic textile dyes using adsorbent synthesized from coffee waste
Source: Sci Rep. 2020 Feb 19;10:2928. doi: 10.1038/s41598-020-60021-6 (PMC7031400; doi:10.1038/s41598-020-60021-6)
Supplement: Supplementary file 1 — Supplementary Materials. [file 41598_2020_60021_MOESM1_ESM.docx]

**Effective removal of anionic textile dyes using adsorbent synthesized from coffee waste**

Syieluing Wong^a^, Nawal Abd Ghafar^a^, Norzita Ngadi^a,^*, Fatin Amirah Razmi^a^, Ibrahim Mohammed Inuwa^b^, Ramli Mat^a^, Nor Aishah Saidina Amin^a^

*^a^* School of Chemical and Energy Engineering, Faculty of Engineering, Universiti Teknologi Malaysia, 81310 Skudai, Johor, Malaysia

^b^ Department of Industrial Chemistry, Kaduna State University, Kaduna, Nigeria

**Corresponding Author:** Tel: +6075535480, Fax: +6075581463, Email address: norzita@cheme.utm.my (N.Ngadi)

**Supplementary Information**

**Section S1 Fitting of adsorption data to isotherm models**

The Langmuir model assumes formation of monolayer by the adsorbate molecules at homogeneous sites on PEI-CW. The linearized Langmuir isotherm are expressed in Eq. (S1)(Al-Othman et al., 2012):

$$\frac{C_{e}}{q_{e}}=\frac{C_{e}}{q_{m}}+\frac{1}{K_{a}q_{m}}$$

(S1)

where C_e_ is the equilibrium dye concentration (mg/L), q_e_ is the adsorption capacity at equilibrium (mg/g), q_m_ is the maximum adsorption capacity (mg/g) and K_a_ is the isotherm constants for Langmuir (L/mg). On the other hand, Freundlich isotherm model postulates multilayer adsorption process on heterogeneous adsorbent surface that possesses dissimilar available sites with distinct adsorption energies. The linearized Freundlich model is expressed in Eq. (S2):

$$\ln q_{e}=\ln K_{f}+\frac{1}{n}\ln C_{e}$$

(S2)

where K_f_ (mg/g) is the model constant and 1/n is the heterogeneity factor associated with the sorption capacity.

According to Eq. (S1), the values of C_e_/q_e_ was plotted against 1/q_e_ for RB5 (Fig. 8(a)) and CR (Fig. 8(b)) to determine the corresponding maximum adsorption capacities (q_m_, from the intercept) and isotherm constants (K_a_, from the slope) respectively. Similarly, according to Eq. (S2), a graph of ln q_e_ vs ln C_e_ was plotted for adsorption of RB5 (Fig. 8(c)) and CR (Fig. 8(d)) to determine the values of model constant (K_f_, from the intercept) and n (from the slope) respectively.

**Section S2 Fitting of adsorption data to kinetic models**

For pseudo-first-order kinetic model, the adsorption rate is proportional to the first power of the concentration. The linearized equation for the model is shown in Eq. (S3) (Naushad et al., 2016a).

$$\ln\left( q_{e}-q_{t} \right)=lnq_{e}-\frac{k_{1}}{2.303}t$$

(S3)

where q_t_ is the adsorption capacity at instant time (mg/g), k_1_ is the rate constant of pseudo-first-order kinetic model (min^-1^) and t is the contact time (min). From the linearized equation of pseudo-first order kinetic model, ln (q_e_–q_t_) was plotted against t to determine k_1_ and predicted/theoretical q_e_ (from slope and intercept) respectively.

On the other hand, pseudo-second-order kinetic model is related to the role of chemisorption as the rate-controlling step for the adsorption process. The linearized equation is displayed in Eq. (S4).

$$\frac{t}{q_{t}}=\frac{1}{k_{2}q_{e}^{2}}-\frac{1}{q_{e}}t$$

(S4)

where k_2_ is the rate constant of pseudo-second-order kinetic model (g mg^-1^ min^-1^). From the linearized equation of pseudo-second order kinetic model, a graph of t/q_t_ is plotted against t to determine k_2_ and predicted/theoretical q_e_ (from slope and intercept) respectively.

In order to determine the role of intraparticle diffusion in the adsorption mechanism, the adsorption data were also fitted to intraparticle diffusion model as shown in Eq. (S5):

$$q_{t}=k_{int}t^{0.5}+C$$

(S5)

where k_int_ (mg g^-1^min^-1^) is the rate constant of adsorption capacity and C (mg/g) denotes the boundary layer effect.

Fig. 9(a) shows the plot of ln (q_e_-q_t_) vs t, while Fig. 9(b) shows the plot of t/q_t_ vs t for adsorption of RB5 and CR dyes respectively.

**Section S3 Fitting of adsorption data to thermodynamic models**

The value of ΔGº was calculated using Eq. (S6) (Naushad et al., 2016b).

$$\Delta G^{\circ}=-RT lnK_{C}$$

(S6)

$$K_{C}= \frac{C_{s}}{C_{e}}$$

(S7)

where ∆G° is the change in Gibbs energy (kJ/mol), R is the ideal gas constant (8.314 J/mol K), T is the adsorption temperature (K), K_c_ is the equilibrium constant calculated using Eq. (S7) and C_s_ is the equilibrium dye concentration on adsorbent (mg/L). The values of ΔSº and ΔHº were determined using Van’t Hoff equation represented from Eq. (S8).

$$\ln K_{c}=\frac{\Delta S^{\circ}}{\Delta R^{\circ}}-\frac{\Delta H^{\circ}}{\mathrm{RT}}$$

(S8)

where ∆S° is the change in entropy (J/mol K) and ∆H° is the change in enthalpy (kJ/mol). From the Van’t Hoff equation, ln K_c_ vs 1/T was plotted to determine ΔS^o^ and ΔH^o^ from slope and intercept respectively.
